# “It’s a matter of building bridges…” – feasibility of a carer involvement intervention for inpatients with severe mental illness

**DOI:** 10.1186/s12888-019-2257-6

**Published:** 2019-09-03

**Authors:** Justina Kaselionyte, Maev Conneely, Domenico Giacco

**Affiliations:** 1 Unit for Social and Community Psychiatry, (WHO Centre for Mental Health Service Development), Queen Mary University of London, Glen Road, London, E13 8SP UK; 2 East London NHS Foundation Trust, London, UK

**Keywords:** Carers, Family, Friends, Inpatient care, Psychosis, Severe mental disorders

## Abstract

**Background:**

Family and friends (carer) involvement in the treatment of people with mental illness is widely recommended. However, the implementation remains poor, especially during hospital treatment, where carers report being excluded from care decisions.

**Methods:**

We developed structured clinical procedures to maximise carer involvement in inpatient treatment. The aim of this study was to test their feasibility across four inpatient wards in East London and explore experiences of the participants. The intervention was delivered by clinicians (social therapists, nurses and psychiatrists) who were trained by the research team. Thirty patients and thirty carers received the intervention and completed research assessments and qualitative interviews after the intervention. 80% of the patients were followed up after six weeks of admission to complete quantitative questionnaires. Six clinicians were interviewed to explore their views on the intervention. Thematic analysis was used to analyse qualitative data.

**Results:**

The intervention was found to be feasible to be delivered within the first week of admission in more than a half of the patients (53%) who provided consent. The main reasons why the interventions was not delivered in the remaining 47% of patients included staff or carers not being available, withdrawal of consent from the patient or patient being discharged prior to the intervention. Two themes were identified through thematic analysis. The first captured participant experiences of the intervention as facilitating a three-way collaborative approach to treatment. The second covered how patients’ mental states and practicalities of inpatient care acted as barriers and facilitators to the intervention being implemented.

**Conclusions:**

Carer involvement in hospital treatment for mental illness is more difficult to implement than is commonly thought. This study has shown that a simple structured approach can facilitate a trialogue and that patients, clinicians and carers appreciate this approach to care. Our intervention provides clear and simple manualised clinical procedures that clinicians can follow. However, even the implementation of such procedures may be challenging in the absence of wider organisational support. The involvement of senior managers and clinical leaders might play a key role in overcoming barriers and support front-line clinicians to prioritise and implement carer involvement.

## Background

Carers are defined as family members or friends of people, who provide unpaid support to people with health conditions [1]. Research has shown that carer involvement has clinical benefits in the treatment of people with severe mental illness. It improves adherence and clinical outcomes and reduces the need for re-hospitalisation [2, 3]. Based on this evidence, UK mental health guidelines and policies [4, 5] recommend carer involvement in the treatment of people with mental illness across their care pathway [1, 6, 7].

Despite support from evidence and policy, carer involvement in the treatment of people with severe mental illness is generally variable and poorly implemented [8, 9]. Yet, research on the implementation of carer involvement is lagging behind research into its effectiveness. A review by the Health Care Commission [8] found that only 20% of mental health trusts were scored as meeting national standards for carer involvement. According to this review, 30% of service user care records did not include a named carer and only one third of staff were trained in supporting families.

A recent report by the Care Quality Commission (CQC) [10] on patient and carer involvement concluded that carer involvement remains inadequate and contributes to the ‘vicious circle’ of poor service user involvement in care. Furthermore, in a CQC carer survey it was revealed that less than 35% carers felt taken seriously or listened to by the mental health services and even fewer were provided with the information and advice they needed [9]. This is echoed by studies exploring carer experiences of hospital treatment for their mentally ill relatives, showing that carers often report being excluded from the treatment process and from discharge planning [1, 11–13].

Thus implementation gap is relevant from a clinical point of view, as previous observational studies on psychiatric hospital care have found that a more positive early experience of care (within 1 week from admission) of patients [14] and carers [15] predicts better longer term outcomes. Qualitative studies have found that a positive experience of care is usually characterised by involvement in treatment decisions [13, 16]. Hence, the need for an intervention starting from the first few days of admission and involving patients and carers in clinical decisions from the outset of the hospitalisation.

In this research project, through a systematic process, we have developed a new intervention to help clinicians maximise carer involvement in the hospital treatment of people with severe mental illness from the first days of their admission. Firstly, we identified the barriers and facilitators to carer involvement in hospital through a systematic literature review [3]. We found that staff training and team work are necessary to build an organisational culture that facilitates carer involvement. Furthermore, the establishment of structured, simple working routines and procedures to support carer involvement could potentially enable clinicians to incorporate it in their practice as balancing clinical responsibilities and workload were identified as barriers. We then carried out a focus group study [17] with patients, carers and clinicians exploring how to enable clinicians to make carer involvement happen in acute inpatient settings. Participants suggested the important components of what carer involvement should entail such as starting early into admission, providing carers with necessary information and involving them in all aspects of care and discharge planning. This informed the development of a training manual and a standardised intervention to enable carer involvement in psychiatric hospital treatment starting from the first days of admission.

The aim of this small scale study was to test feasibility of a simple one session carer involvement intervention in acute psychiatric wards and to explore experiences of patients, carers and clinicians.

## Methods

### Design

A feasibility study was carried out with 30 patients and 30 carers in the year 2017. Assessments included quantitative assessment of feasibility and qualitative interviews with patients, carers and clinicians. The study received favourable opinion from the Essex Research Ethics Committee (East of England) – reference number 15/EE/0456.

### Sampling

Consecutive sampling was used to recruit patients newly admitted to four inpatient psychiatric wards at Newham Centre for Mental Health (NCfMH) of the East London NHS Foundation Trust (ELFT) allowing for any diagnoses of mental disorders. Eligible patients were identified by screening the list of admissions on the electronic records database RiO for each participating ward and were approached by the clinical team.

Patients were eligible if they were within a week from admission, over the age of 18, had a carer (family member or a close friend who provided unpaid support), the capacity to consent to the intervention, and a command of the English language sufficient to express their carer involvement and information sharing preferences and participate in a meeting meaningfully.

Carers were eligible if they were 18 years and older and had a command of English language sufficient to participate in a meeting.

Ward managers and modern matrons of inpatient wards at the NCfMH ELFT were informed of the study and, upon their agreement clinicians working on their words were trained in the intervention. Clinicians included ward managers, charge nurses, staff nurses, junior doctors and social therapists.

### Intervention

At the core of the intervention is a patient-centred and resource oriented approach [18], inspired by available research evidence [3] and the good practice example of the Family Liaison service provided at the Somerset Partnership NHS Foundation Trust [19]. The intervention focuses on identifying and engaging with the social support network of the patient. It can include participants either face-to-face or through a video conference call (i.e. using Skype interface) in situations where the patient’s carer lives far away and is unable to come to the ward.

The intervention was designed to take place within the first week of inpatient admission, as there is evidence from observational studies [14, 20] that this is a critical period in which a better experience of care can predict better clinical outcomes (i.e. reduced re-hospitalisation rates and better quality of life 1 year after discharge).

**Fig. 1:**
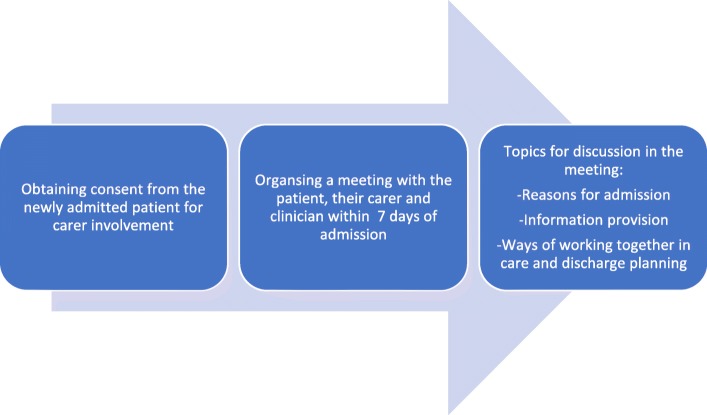
The components of the carer involvement intervention

The intervention comprises manualised standardised procedures for: a) Discussing patient’s consent for carer involvement immediately after they were admitted to the hospital; b) organising a meeting with the patient, their chosen carer and a clinician within 7 days of admission; c) during the meeting discussing reasons for admission, the ways of working together in care and discharge planning as well as providing patients and carers with the information they need. The patient can specify any topics they do not wish to discuss during the meeting at the point of providing consent; these might include their reasons for admission, diagnosis or treatments. The components of the intervention have been summarised in Fig. 1.

The one-hour training provided to clinicians delivering the interventions also includes basic skills for communication in three-way meetings and facilitation of such meetings. Clinicians received a copy of the 32-paged intervention manual for their reference which includes the structure of the intervention, its necessary steps, meeting agenda that needed to be followed with suggested wording which clinicians could use while chairing the meeting. The manual also includes guidelines for communication and meeting facilitation which clinicians could refer back to while preparing for the intervention.

### Procedures

Clinical teams across four inpatient acute wards were trained in the intervention by authors JK and DG. Training was delivered mostly in groups during team away days, and in certain cases where a new member of staff joined the ward team, on a one-to-one basis. Patient and carer representatives with experience of several hospital admissions were involved in some of the group training sessions where they shared their experiences of carer involvement and participated in group exercises which included role plays. Upon completion of the training clinicians received regular supervision (at least two 15 min sessions) and support from the research team during which clinicians were able to ask questions and express any feedback or concerns.

The intervention, rather than being a deviation from best practice guidelines, was a structure intended to implement/facilitate routine practice, therefore informed research consent was not required. Eligible patients were approached by JK and asked: "Do you have someone who supports you (a family member or a close friend)? If the patient identified someone, then they were asked if they wished to invite them to the intervention session. The aim and content of the session were explained to the patient and a consent form was completed specifying the names of carers as well as the patient’s information sharing preferences. If the patient did not provide consent for carer involvement, reasons for declining were recorded.

The researcher contacted the carers and organised the intervention session based on their availability. Interventions were delivered on the wards, in a quiet room (most often a designated family or therapy room) by a trained clinician. In one case where the carer lived abroad, their participation was ensured using videoconferencing on Skype interface.

Once the meeting was organised and both the carer and the patient were present on the ward, the clinician met with them to conduct the intervention and used their time and their facilitation skills to the best of their ability with no influence from the researcher.

### Data collection

After the intervention (mostly on the same day), those patients and carers who gave informed consent for research, met with the researcher to collect sociodemographic information, complete research assessments and qualitative interviews. Patients were administered the following questionnaires: 1) Patient version of the Clients’ Scale for Assessment of Treatment (CAT) [15, 21]; 2) Involvement Indicators Scale [22]; 3) DIALOG scale [23]. Clinical Global Impression (CGI - severity of illness) score [24] was obtained from the patient’s psychiatrist. Carers completed the carer version of the CAT [15, 21].

Individual qualitative interviews were conducted with patients, carers and clinicians exploring their experiences of the intervention and their views on its individual components. A topic guide was used to aid the discussion. Interviews were conducted by JK in a quiet room or patient’s bedroom.

All patients and carers were invited to interviews, whilst clinicians were purposively sampled based on working in different wards, professional backgrounds and amount of experience in working in acute mental healthcare.

Six weeks after admission/intervention, JK contacted patients by phone to collect follow-up data using Involvement Indicators Scale [22], DIALOG Scale [23] and Sign O’Brien Level of Engagement Scale (SOLES) [25]. Follow-up assessments were also conducted in person if the patient happened to be an inpatient on the ward.

If the patient provided consent, information on discharge, follow-ups, diagnosis and further carer involvement was extracted from electronic records on RiO database.

Patients and carers were reimbursed for their participation in the assessments and interviews with shopping vouchers (£5 remuneration voucher for each of assessment).

Throughout the study, the following feasibility measures were also collected: 1) Number of patients who were not eligible or declined participation in the intervention; 2) Number of carers who could not be approached or did not wish to participate; 3) Number of carers attending the intervention; 4) Length of the intervention; 5) Percentage of patients and carers completing the research assessments and interviews and time required to complete them; 6) Time required to complete the assessments.

Clinicians who delivered the intervention also completed a short questionnaire which included questions on how many times the patient and their carer(s) were approached and how, who attended the meeting and the mode of the meeting (in person, Skype, phone). Clinicians were also asked to write their experiences of facilitating the meeting and note down if further carer involvement was discussed and agreed.

Finally, JK documented her contact and discussions with clinicians throughout the feasibility study noting down any challenges that she observed when supporting clinicians with the implementation of the intervention.

### Analysis

Descriptive statistics were used to summarise the feasibility measures of the intervention and demographic participant data.

The interviews were audio-recorded and transcribed verbatim, removing any identifying information. Transcripts were analysed using thematic analysis [26]. NVivo software was used to aid the analysis. JK completed the initial coding of the transcripts and then organised the codes into sub-themes and themes. MC independently coded 20% of transcripts and checked the congruency of the themes. Any disagreements and final themes were discussed by the research team (JK, MC and DG).

Simple content analysis [27] approach was used to analyse and summarise clinician feedback from the intervention recording forms and the researcher’s observations.

### Reflexivity

Research team members have a background in different disciplines and are mental health researchers. JK is a social scientist, MC is a research psychologist and DG is an academic and clinical psychiatrist.

All authors have an interest in acute mental health care and carer involvement in psychiatric treatment of people with serious mental illness. They all have experience of participating in previous qualitative research.

It could be also noted that the authors believe in the clinical effectiveness of carer involvement and their stance is that a patient-led approach and carer involvement can improve experience and quality of care. This may have influenced the results but the paper has been discussed paper in a larger peer group of mental health researchers to receive additional feedback.

## Results

### Participant characteristics

**Fig. 2:**
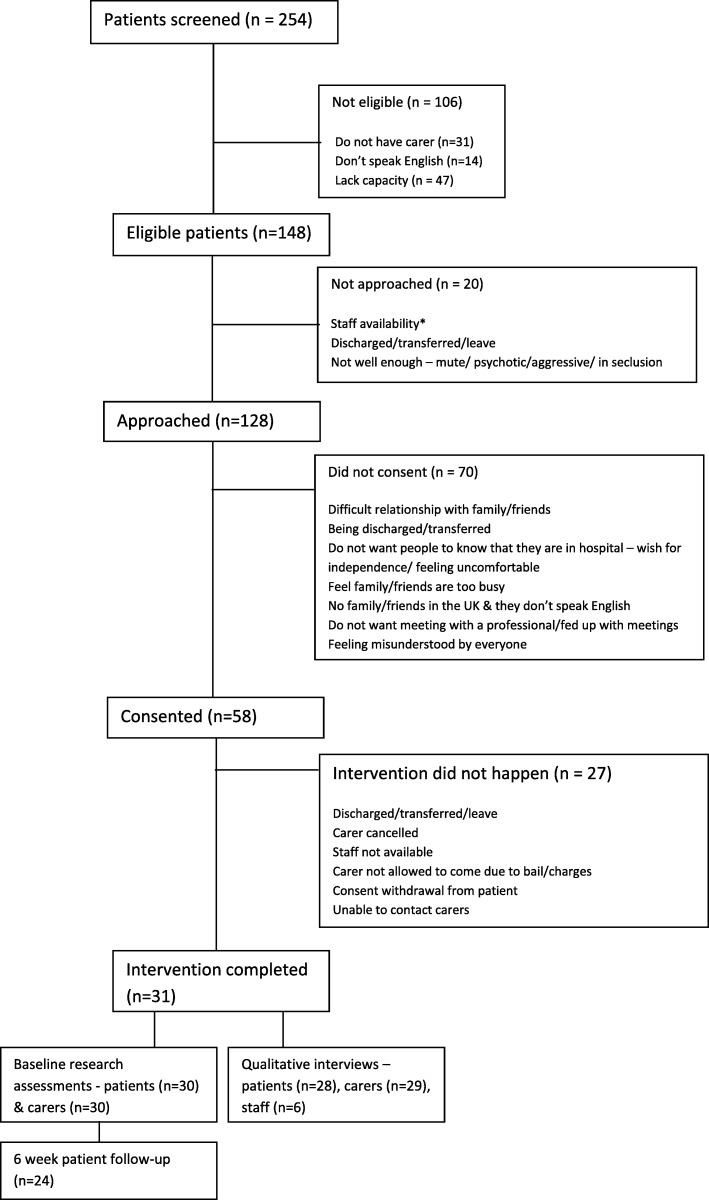
Flow chart diagram of the screening and consent process

Across four acute inpatient wards, 254 newly admitted patients were screened for eligibility (see Fig. 2). Fifty eight percent of patients (*n* = 106), were not eligible for the research because they lacked capacity to communicate their preferences for carer involvement (*n* = 47), did not have a carer (*n* = 31) or did not speak English (*n* = 14). Twenty eligible patients were not approached due to them being discharged on the same day or not being available on the ward (e.g. being placed in seclusion or transferred to an intensive care ward).

Of those approached, 58 patients (45%) provided consent for their family member or a friend to be contacted and invited for the intervention session. The main reason why consent was withheld by some patients was the difficult or broken relationship that they had with their relatives and/or friends (*n* = 14). There were occasions, where patients did not want anyone to know that they were receiving treatment in a psychiatric hospital (*n* = 9) or felt that their carers were too busy with their work and family commitments and did not want to burden them any further (*n* = 9) amongst other reasons (please refer to Fig. 2).

Intervention was completed with 31 patients - 53% of those who consented (*n* = 58). The planned interventions that did not go ahead were due to staff unavailability to conduct the intervention (*n* = 4), carer approachability and availability (*n* = 6), consent withdrawal from the patient (*n* = 2) or patient’s discharge prior to the intervention (*n* = 10) or the patient going absent from the ward (*n* = 2). There were also three occasions, where the patient’s chosen carer was not able to participate due to bail charges imposed on the patient.

The majority of patients who took part in the feasibility study were female (*n* = 20, 66.7%), single (*n* = 22, 73.3%) and born in the United Kingdom (*n* = 19, 63.3%). The mean age of patient participants was 34.8 (SD – 12.3). Carers were predominantly female (n = 19, 63.3%), married (*n* = 13, 43.3%) with the average age of 47.3 (SD = 16.8). Six clinicians with the average 10.9 years of experience in mental health (SD = 12.9) took part in qualitative interviews. The main sociodemographic and clinical characteristics of the participants were summarised in Table 1.

**Table 1:** Sociodemographic and clinical characteristics of participants

Patients (*n* = 30)	n (%) or mean (SD)
Age (years)	34.8 (12.3)
Gender (% female)	20 (66.7)
Marital status (% single)	22 (73.3)
Country of birth (% UK)	19 (63.3)
Education (% tertiary)	17 (56.7)
Accommodation (% independent)	25 (83.3)
Living with partner/family (%)	20 (66.7)
Employment (% unemployed)	13 (43.3)
CGI score	4.1 (1.0)
Index admission days	16.8 (13.2)
Re-admission within 6 weeks	5 (16.7)
Remained inpatient within 6 weeks	4 (13.3)
Involuntary admission (%)	14 (46.7)
Follow-up attended (%)	20 (66.7)
Diagnosis	
Psychotic disorder	13 (43.3)
Mood disorder	12 (40.0)
Other	4 (13.3)
Missing	1 (3.3)
Carers (*n* = 30)	n (%) or mean (SD)
Age (years)	47.3 (16.8)
Gender (% female)	19 (63.3%)
Marital status (% married)	13 (43.3%)
Country of birth (% UK)	12 (40.0%)
Education (% tertiary)	18 (60.0%)
Employment (% employed FT or PT)	14 (46.7%)
Relationship	
Parent	13 (43.3%)
Sibling	6 (20.0%)
Partner	4 (13.3%)
Child	3 (10.0%)
Uncle	1 (3.3%)
Friend	3 (10.0%)
Duration of care	
First contact (2 weeks or less)	6 (20.0%)
1 Year or less	4 (13.3%)
5 Years or less	6 (20.0%)
More than 5 years	14 (46.7%)
Clinicians (*n* = 6)	n (%) or mean (SD)
Age (years)	39.8 (18.1)
Gender (%female)	3 (50)
Years working in inpatient care	4.2 (5.4)
Years working in mental health	10.9 (12.9)

Thirty patients and 30 carers gave informed consent to complete research assessments and qualitative interviews. Six clinicians were purposively sampled for qualitative interviews.

### Intervention delivery characteristics

Seventy-one staff members across four wards were trained in groups and individually. Training session was approximately 1 hour long and clinicians also received a manual for the intervention. There was a good spread across different disciplines across the sample (Charge nurse– 26.1%; staff nurse - 26.1%; social therapists – 30.4%, doctors - 17.4%). Of these, 23 clinicians delivered the intervention to 31 patients and their carers.

We recorded how many attempts were made to approach patients and contact carers to invite them to take part. The majority of the patients (*n* = 30, 96.8%) were approached only once. Seven carers were contacted more than once 24 (77.4) due to them being busy and unable to answer the phone or a text message.

The average length of session was 28.8 min (SD-14.4). More than a half of the interventions happened in the first 3 days of patient’s admission. Thirty sessions were completed in person and one over Skype videoconferencing with the patient and the clinician being present on the ward and the carer joining in from a country abroad. Intervention characteristics have been summarised in the Table 2.

**Table 2:** Intervention characteristics

Intervention session (*n* = 31)	N (%) or mean (SD)
Organised in 1–3 days	16 (51.6%)
Mean length of session (mins)	28.8 (14.4)
SU approached once (%)	30 (96.8)
Carer contacted once (%)	24 (77.4)
Delivery mode (in person %)	30 (96.8)
One carer per session (%)	21 (67.7)

### Follow-up information

We achieved 80% follow up rate with patient participants. The remaining 20% were lost to follow up due to being uncontactable, too unwell to provide informed consent or not interested in participating anymore and declining to take part. Follow up assessments with patients were conducted on the phone in the majority of cases – 70.8%. Others took place on the inpatient wards.

### Clinician feedback from meeting recording forms

Clinicians provided their feedback on facilitating the meeting by completing a short questionnaire after the meeting ended. Recording forms were completed for all 31 interventions that took place during the study. Overall clinicians felt that the facilitation of the meeting following the manual was straightforward and shared positive experiences of discussing ways of working in collaboration with patients. They reported that participants engaged well and felt relaxed during the meeting. Clinicians also felt that the meeting allowed them to agree with carers their regular engagement in the care planning of the patient (i.e. ward round attendance).

However, in three cases clinicians reported difficulties in manging the dynamics between the patient and their carer in the room or facilitation being difficult in terms of keeping to the agenda or the patient not feeling well enough to participate.

### Researcher’s notes on intervention implementation

JK notes captured the difficulties in making the intervention happen after the consent from both the patient and carer was obtained and meeting time and date was organised. Often the intervention did not go ahead due to the clinician being unavailable and in most cases the carer would already be present on the ward for the meeting but would need to return home without having the intervention as no one on that ward was able to meet with them.

JK also documented other barriers for the intervention delivery that were encountered on wards such as the patient’s legal status, lack of facilities, carer availability as well as the patient being discharged before the day of the intervention.

“Intervention did not happen due to the nurse who agreed to do the intervention going off the ward and, after waiting for him for an hour; the patient withdrew her consent and asked the carer and the researcher to leave”.

“The carer arrived at 8pm. Clinician 9 who previously agreed to do the intervention was not on shift anymore. Clinician 8 who was trained in the intervention was working instead of him. She complained that carer arrived after visiting hours and was very reluctant to do the intervention. I have to spend approximately 15 minutes trying convincing her to meet with the patient and her friend explaining that the patient does not have anyone close in this country and her close friend was very keen to be involved but the only time he could come was in the evening after he finished work as he had a busy schedule. Eventually she agreed but was very unhappy about that.”

“I convinced clinician 4 to meet with patient’s daughter. However, she arrived with two children who could not be allowed on the ward. Nobody knew that she'd bring children and the carer did not know that they were not allowed. I found a room downstairs for them to wait but soon it transpired that the patient could not leave the ward due to her legal status so the intervention could not happen. Meeting re-scheduled.”

“The patient gave consent to do the meeting with his girlfriend. When I checked with a member of staff about it, it transpired that the patient had beaten girlfriend and staff were reluctant to get involved until the police informs them how to proceed. We agreed to postpone it until the situation is clarified. A few days later, the patient went on planned leave.”

### Thematic analysis findings

**Fig. 3:**
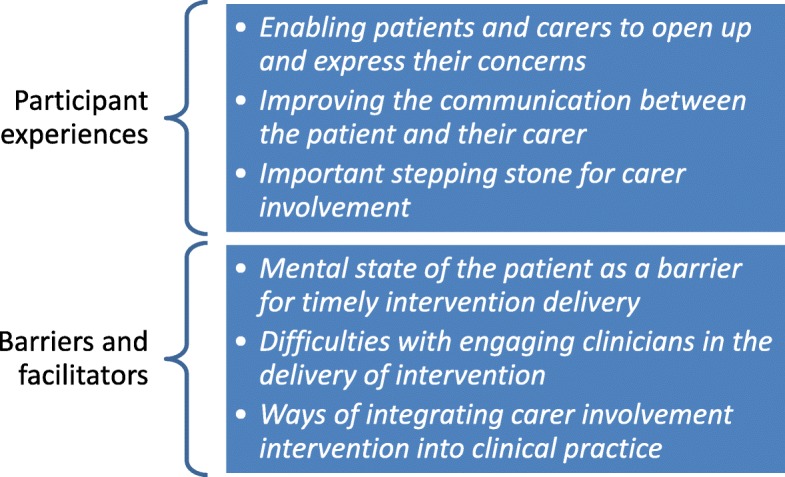
Themes and subthemes from the thematic analysis

Two themes emerged from the data: participant experiences of the intervention and the barriers and facilitators encountered in delivering the intervention. The first theme includes data from all three participant groups – patients, carers and clinicians while the second one stems from the data collected in the clinician interviews. The themes and their subthemes are presented in Fig. 3.

### Participant experiences of the intervention

#### Enabling patients and carers to open up and express their concerns

Both patients and carers who took part in the study felt that the informal and relaxed atmosphere during the intervention session allowed them to open up and express themselves, sharing their stories and concerns. The intervention session was often contrasted to a clinical review meeting that was perceived as formal, with medical jargon, causing anxiety and often lacking the space for patients and carers to voice concerns.

“They were, so happy and relaxed, and at the end of the meeting, they thanked me a lot for the time, for doing it that way, because she said, “oh, it’s different than the ward round”, and, you know, it’s more something more intimate I would say, and it made them feel very relaxed.” (Clinician 1).

“It was quite an informal meeting which was really nice. It’s not… you don’t get bombarded with medical terms and it’s very friendly and relaxed. So that’s a really good thing as well… you feel comfortable.” (Carer 3)

Furthermore, participants felt that they were not pressured to share things that they were uncomfortable sharing or answer questions that they did not feel like responding to.

Another important aspect of the intervention that allowed especially patients to feel at ease and express their feelings was the fact that there was only one clinician and one or two carers in the room which felt less intimidating in comparison to other meetings that patients attended on the ward:

“When the meetings are really big it gets a bit like, “oh my gosh!” you might not wanna speak (…) So this is good that it’s less people. You don’t feel like you need to hide anything.” (Patient 10)

“Yeah, with less people, coz it’s so… it’s hard really with so many people.” (Patient 2)

The facilitation style of the meeting also played an important role in creating an informal and relaxed atmosphere during the intervention session. Clinicians adopted a non-judgemental approach, used active listening skills and made sure that both parties had equal opportunities to express themselves and validating their feelings.

“I think [clinician] did that well and he asked my opinion first then he asked my parents’ opinion. So obviously as a service user I would see everything differently to how my parents see it.” (Patient 10)

“I was telling my boyfriend off in front of the doctor realistically and the doctor wasn’t judgemental at all. He just said, “okay, I do understand your frustration” and you know he explained why I see this the way I see it and why [Patient name] sees the way he sees it. So yeah, we had quite a meaningful discussion.” (Carer 21)

Carers in particular, felt listened to and reported being able to ask questions about their relative’s care that they were concerned about. This was perceived as a positive change in service provision that they had not experienced before during their contact with clinicians. Patients also felt that the intervention session gave their relatives a forum to voice their concerns which was lacking in clinical meetings on the ward.

“It was very beneficial because I felt the last time all these issues about not being seen urgently enough and her mental health deteriorating (…) - I felt all that was inside and I had not been able to express it. So it was actually good being able to express those issues.” (Carer 22)

“It is a good thing because the ward rounds are so short sometimes parents don’t get an opportunity to ask questions that they might want answers to. So it’s like an added thing where they’ve got a little bit more time to sort of discuss things.” (Patient 12)

### Improving the communication between the patient and their carer

Carers felt that the intervention helped them to improve the communication with the patient and appreciated that the intervention offered them a space to be “clear and open about everything” (Carer 13) which made it possible to possibly improve their relationship. Often patients disclosed information that was not previously discussed with the carer, in particular, the reasons for admission to hospital, which carers felt was helpful.

“Lots of things came out of it that I didn’t know before. (…) It was very beneficial.” (Carer 20)

“It was good for my dad to hear what brought me in coz before I didn’t really tell him (I: So that was the first time that he actually heard?) Yeah, why I was put in here.” (Patient 3)

These discussions were not without difficult emotions, which clinicians had to manage in the room. However, both patient and carer participants admitted that although it was not easy to share their emotions in the room, it made them feel better and cleared the air between them.

“It was very emotional and you know him saying nobody cares about him when I give him examples of when I showed my care and I said, “what do you think about it?” Just like, “I do admit you cared for me”. You know the clinician was saying probably he didn’t want to be judged by you and I said, “okay, that’s fine”. (…) So yeah, it was quite an interesting conversation.” (Carer 21)

“Yes, it’s good, it’s literally good, it’s good value meeting what you have. (…) it was emotionally hard meeting as well and I could see that I could feel those emotions before probably I couldn’t and I stayed cold as I wanted. It helps to evaluate yourself and see other people so.” (Patient 20)

The ability to discuss often pressing and difficult issues was given by the intervention providing the space to include all three parties in the discussions – the patient, carer and clinician, which was welcomed by the participants who felt this was a transparent way to talk about the current situation and helped maintain their relationship.

“It’s good to have the patient there as well especially with the ones that suffer with paranoia which [patient] does, so I like to do everything with [patient] there because then she knows I’m not colluding with anyone or trying to keep her in for longer than she needs to be. So I think it is good that both parties are in meetings.”(Carer 3)

“That’s good - obviously you get to know my view and my parent’s view at the same time. (…) You can see the different how I feel and how they feel about the same kinda thing.” (Patient 10)

However, a view was also expressed by carers about having a separate meeting with the clinician before or after the intervention meeting as carers often did not feel comfortable discussing certain issues in the presence of the patient due to the content of these discussions being potentially upsetting to the patient.

“What I did mention was having individual meetings, like just five ten minutes with [patient] on his own without me being there and then just me five ten minutes if I need to say anything. Coz I might say something that’s gonna upset him and then I’m restricted in saying something, coz I don’t wanna upset him or he might wanna say something that he feels that I’m gonna get upset. So it’s nice to have either me or him not there at each time.” (Carer 2)

“If I tell to doctor that she’s not ready to go home yet when she’s that sick, she’d go crazy and think I’m going against her. (…) when she’s really sick like that, you couldn’t say that in front of her, because she’d go crazy and think you’re like the doctor trying to keep her in jail. (…) So you need that space sometimes to see the doctor and say well actually I couldn’t say this in front of her” (Carer 6)

### Important stepping stone for carer involvement

The intervention provision was seen as an important stepping stone for carer involvement by the participants. Carers in particular, emphasised the urgency of carer involvement early into admission which the intervention offered.

“I think it’s invaluable because it’s a matter of building bridges, it’s also dispelling any kind of misapprehension between carer and loved one. It creates a working atmosphere that staff, carers and the loved one can, or service user can work within. It also enables the carer plus the service user to realise that staff are on your, on your side as well. (…) You get that we’re here to support you both.” (Clinician 9)

“All you know your relative is sick and they are coming to hospital and then you don’t know what’s going on. So you can come and visit. It’s so important to let people know what’s going on. So I think it’s very important to have these meetings, yeah.” (Carer 6)

Carer participants also reflected on their previous negative experiences of being excluded from care planning when their relative was in psychiatric hospital. They appreciated that this time they were invited to meet with a clinician and their relative, provided information and felt that this reduced their anxieties and facilitated their future involvement in their relative’s care. This was also reported by the patients who could compare this to their previous experience elsewhere.

“I’m quite impressed really. (…) just really appreciate that you’re taking the time to do it really coz that’s of my experience and maybe [area name] is different or I think it’s quite unusual. (…) I just wish other hospitals in other parts of the country would do something similar really.” (Carer 16)

“I think it was helpful in some form and I think that what you’re doing is very useful coz I’ve had other hospital admissions in other places and they don’t offer any of that to family and friends. They have no involvement in their care and I think that’s very detrimental and I think that with the research that you’re doing hopefully other people will do the research and give their opinions and hopefully then you can be able to work with family and friends together with patients for the best care.” (Patient 16)

### Barriers and facilitators of delivering the intervention

#### Mental state of the patient as a barrier for timely intervention delivery

A concern was expressed by clinicians about newly admitted patients being too unwell to engage, provide their preferences for carer involvement and informed consent to take part in the intervention itself. It was also felt that this would hinder carer involvement as the patients have the tendency to simply reject the invitation to involve their carers when they come to the hospital. Therefore it was suggested to consider flexibility in terms of the timeframe of the intervention to prevent those patients who are still unwell from missing out on the intervention. Furthermore, even if consent was provided, the quality of such meeting would suffer if the patient were feeling too distressed that day.

“Sometimes they don’t agree because they are still acutely unwell, and they don’t want to talk, they don’t want to say absolutely nothing, and sometimes that happen, that they are completely mute, maybe for a week, just hiding themselves in their room, and there is no interaction also with ward staff, with doctors, so that can be barrier for someone, that they are still so unwell that they don’t, cannot give any kind of consent (…) you should consider, also, to extend for someone (…) if you set the timeframe to one week, you can pick some patient and not others.” (Clinician 1)

#### Difficulties with engaging clinicians in the delivery of intervention

Busy ward environment, emergencies and competing clinical priorities were pointed out by clinicians as a primary barrier for the intervention delivery. Staff shortages on the wards were often the reality which affected the ability of staff to deliver the intervention within the timeframe of 7 days.

“It just depends if we’re just because sometimes we’re so so busy that it’s hard to, to fit it in.” (Clinician 21)

“So obviously if there’s a staff shortage, it might be really difficult” (Clinician 50)

Clinicians felt that even when they had committed to deliver an intervention, in the event of an emergency they could be called to attend to it and therefore would be unable to meet with the patient and their carer. Forward planning and using protected time were suggested as facilitators that could help to ensure that there are clinicians to deliver the intervention.

“Maybe a bit of forward planning so the person is admitted that day and I know I’m in tomorrow. Make forward plan for tomorrow and make sure it’s in the diary and everyone knows that for that protected time.” (Clinician 50)

However, staff availability to deliver the intervention was also affected by their “lack of interest” (Clinician 9) or motivation to adopt a new intervention and failing to see carer involvement as a priority. It was suggested that a change in organisational culture may be required to ensure carer involvement in hospital.

“I know that the staff say that they are extremely busy on the ward, but anything that’s changed the normal routine is a bit, you know, they are biased, because when you know that that is the routine, you have to change something, (…) and that is why maybe it has been a bit difficult to find the staff that gives some collaboration.” (Clinician 1)

Clinicians also suggested strategies for motivating staff to incorporate the intervention in their daily clinical practice such as ensuring that all staff are informed about the evidence-based effectiveness of the intervention. Others felt that clinicians require some time to adapt to the newly introduced intervention.

“I think we just need to sort of I suppose change the culture on the ward, just get it involved, make sure that this is something that’s really really important. (…) And then just I suppose making sure that everyone knows what benefit this can have and then that should really keep it going.” (Clinician 50)

“The main thing is that we just, when you roll it in and then we get used to it and then we get on with it. (…) It’s just human. When you are learning something for the first time you wonder can I do it? But once you get used to it that’s it. (…) if somebody does it the first time likely they will do it a second time. It’s that, just that first time, first time.” (Clinician 56)

### Ways of integrating carer involvement intervention into clinical practice

Clinicians felt that support from leadership was paramount in ensuring that carer involvement procedures become integrated in every day clinical practice on psychiatric wards. Team leads were seen as potentially motivating and supervising staff in one-to-one meetings ensuring that they are following the carer involvement procedures and giving them confidence to deliver these.

“I think if you’ve got (…) the team leaders on board who can really push it and can make sure that everyone’s up for it and like engaging in it then I think it could be quite successful (…) (…) So it’s something that I can mention in my supervision. (…) some people might just not have the confidence to do that. (…) I could always go in there and support them on the first couple of those think it’s just thinking about how we can support the staff so that they can go on and do it.” (Clinician 50)

Staff training was also an important facilitator of the carer intervention and training more clinicians was seen as a way to increase flexibility and ensuring that a member of staff is always available to deliver the intervention when the carer comes to the ward. Others felt that having a designated link person would be the most helpful as they would always be available to make carer involvement happen while other clinical staff are being drawn into other clinical priorities.

“That’s why if you train more people then when it’s ad hoc they’re not to rely on the one person then to take or chair or sit in on all these meetings. It’s four or five people on the same shift who could run the same meeting making it as open as possible so that the loved one can feel free to know they can call up today and come in tomorrow.” (Clinician 9)

“I think it’s quite thoughtful to have a designated person to sort of it was sort of like a link person to help us really focus on that area. Have that link with the family as well coz as you see we try and have contact with the family but sometimes when it gets so busy if there’s more thing like priorities sometimes it can go down the list it was really helpful.” (Clinician 50)

More specific practical suggestions were given by clinicians such as including the carer involvement procedures in the admission checklist, allocating designated staff in the rota on a daily basis and including carer visits in the ward diary/ communication book in order to make the whole ward team aware of this.

## Discussion

### Main findings

We found that the simple clinical procedures that we have developed and tested in this feasibility study were feasible to implement within 7 days of the patient’s admission in more than a half of the patients (53%) who provided consent.

Patients were of various diagnoses and had a range of severity of symptoms and yet were able to participate meaningfully in the intervention. Carers sample was also varied and included not only parents, siblings or children of the patient but also wider family such as uncles and close friends.

Positive feedback was received from patients, carers and clinicians who participated in the intervention. The intervention was perceived as an important stepping stone for carer involvement which enabled patients and carers to open up and express their concerns as well as be open with one another which in turn improved their communication. Clinicians in their feedback forms reported that facilitation of the meeting was straightforward and helpful to all three parties. Moreover, the length of the sessions varied, with some of them being as short as 15 min. The clinicians reported that they were able to cover the main agenda items in the manual within the timeframe. The length of the meeting might have been affected by the clinical state of the patient. For example, some patients may only have been able to stay for a limited period of time due to their psychological distress and, hence, the discussion was shorter. However, the positive feedback received from the participants, in particular carers, suggests that however short was extremely beneficial for them and reduced their anxiety about their loved one.

However, we also identified a number of barriers to the implementation of the carer involvement intervention. Half of the interventions that were scheduled with carers were not delivered as planned. This mainly happened due to clinicians not being available to facilitate the session. The difficulty engaging clinicians into delivery of the intervention was also one of the subthemes identified from the participant interviews and also supported by the observational data from the lead author. While clinical demands and understaffed wards played a role, the low motivation to involve carers may be related to the organisational culture that requires a change. Strong support from the ward leadership was suggested as paramount in motivating and supervising clinicians in integrating the carer involvement procedures into daily ward practice.

Patients’ mental state was perceived as a barrier to the delivery of the intervention by clinicians. Indeed, half of the newly admitted patients were not eligible due to the lack of capacity to communicate their wishes during their first week of admission. However, this should not preclude the option of offering the intervention early into admission, as carer involvement is regarded to be good practice and unlikely to cause significant harm. Starting early does not seem a significant barrier either. Over a half of the completed interventions (51.6%) were conducted in the first 3 days of the patient’s admissions.

### Strengths and limitations

This is the first feasibility study to test a carer involvement intervention in inpatient psychiatric care settings. The intervention was implemented on four different acute inpatient wards including a triage and assessment ward with rapid patient turnover along with more traditional acute psychiatric wards. The study was not restrictive in terms of diagnosis or clinical symptoms and thereby was able to capture patient views across a diverse sample, representing the breadth of inpatient treatment. We have provided training to whole clinician teams to ensure their availability to deliver the intervention and also involved various disciplines in the delivery of it.

However, the study was conducted in only one hospital – a mental health centre in East London and, the organisational and governance arrangements may not be generalisable to other locations in the United Kingdom or abroad. The study is also limited by excluding non-English speaking participants and those under 18 years of age from the sample. Furthermore, we only recorded participants’ country of birth and did not collect the information on their ethnicity which may have provided important considerations given the geographical location of the hospital and the national profile of inpatients. The backgrounds and interests of the researchers could have influenced the interpretation of the data. However, the bias of the interpretation was reduced by the wide range of the disciplines in the research team and rigorous methodology. Furthermore, we did not formally assess clinician’s fidelity of delivering the intervention. However, we asked for their feedback on the intervention delivery in a less structured way - through a short questionnaire completed shortly after the intervention. Moreover, we were able to gather the information of what agenda items were covered from the interview with the patient and the carer. Interviews were conducted as soon as possible after the intervention to prevent recall bias.

Finally, the research team was actively involved in supporting the clinicians in screening and approaching patients and organising the intervention meetings by phoning the carers and also making sure that the scheduled intervention meetings were going ahead which often involved convincing trained clinicians to deliver the intervention. This must have contributed to the improved rates of delivered interventions. However, one may argue that logistic and organisational support is important for many interventions delivered in real world health care settings. Moreover, this close involvement allowed an in-depth observation of the ward culture and procedures which helped make sense of our results.

### Comparison with literature

Available audits and reports from the Healthcare Commission [8] and Care Quality Commission (CQC) [9, 10] demonstrate poor rates of carer involvement in the treatment of people with mental illness. In our study, we achieved 53% implementation rate of our intervention within 7 days of the patient’s admission, which is higher than the current national average of 20% that was found by the Healthcare Commission [8]. Furthermore, their review reported only one third of staff trained in family involvement. Our study took a holistic approach and trained the whole staff teams that were available to deliver the intervention, which may also have contributed to the higher implementation rate. Finally, the Healthcare Commission [8] identified that one third of patient records did not have a named carer. In our study, clinicians approached all newly admitted patients and asked for their preferences for carer involvement. Indeed a systematic approach towards this may help ensure that no one is missed out and all patients who consent to carer involvement have a named carer recorded in the records. Most importantly, the implementation of even such a simple intervention required during our study the committed work of a researcher in order to support clinicians in organising logistic aspects. This is likely to have played an important role in increasing participation of carers, patients and clinicians. The need for this logistic support should be considered when implementing similar interventions.

We found that even a simple one-session intervention can boost the positive carer feelings towards the service and care their relative is receiving. The carer participants in our study felt that they were able to voice their feelings and concerns and perceived the intervention an important step towards involvement which often was reported as not happening previously.

This is in line with the findings of Radcliffe [27] who found that emotional support, validation of carers’ feelings and improved communication between carers and mental health professionals was very much valued by families. Moreover, this is frequently expressed as an expectation from services by carers [11] which is often not met by acute psychiatric services [28].

It can be argued that our findings about the barriers and facilitators for the intervention delivery are in line with the existent literature. A systematic review by Eassom and colleagues [3] found that carer involvement in the treatment of mental illness is often impeded by competing clinical responsibilities, organisational culture and paradigms as well as the lack of shared team commitment for family work. They also identified that support and supervision from the leadership who would endorse carer involvement and in turn help change the culture of the service was paramount, which was also suggested by the participants in our study.

### Clinical implications and further research

Barriers such as clinician availability and willingness to deliver the intervention could be overcome by involving ward leadership to provide support and supervision for this and implementing simple strategies on the ward level, such as introducing protected time for carer involvement meetings which could be included in the ward diary to make the whole team would be aware of this.

To ensure that all clinicians are trained in a time efficient way a short training module which or an online delivery of training could be a way forward for scaling up across different services.

Finally, we feel the need to acknowledge that carer involvement is a complex phenomenon and that there are some patients, carers and clinicians which will be more difficult to engage in such initiatives. Their engagement requires new thinking, research and strategies such as not only training all clinicians on the ward but concurrently carrying out awareness raising programmes and workshops to ensure buy-in of senior managers. Furthermore, innovative strategies to ensure carer participation might be enabled by skype or other systems for remote communication. Our experience, although limited to one case, shows that it is possible to implement this.

Future research should focus on testing these procedures in different contexts including remote and rural areas of the country and understand whether there is a need for adaptation.

## Conclusion

Even short carer involvement interventions can be challenging to implement on acute inpatient wards. However, they may be worthwhile and beneficial given the overwhelming positive experiences reported by the patients and carers who took part in the three-way collaboration. An initial meeting with the patient, their carer and a clinician that takes place in the first days following admissions could be the stepping stone that provides the opportunity for connection between what happens within and outside the hospital doors and could lead to improved patient outcomes.

## Data Availability

The dataset supporting the conclusions of this article is stored securely in the Unit for Social and Community Psychiatry of the Queen Mary University of London according to UK Information Governance standards. Any interested researcher should contact Dr. Domenico Giacco (d.giacco@qmul.ac.uk). A data sharing agreement will be needed in case researchers outside of the primary research team will request to access these data.

## Abbreviations

CAT: Clients’ Scale for Assessment of Treatment
CGI: Clinical Global Impression
CQC: Care Quality Commission
ELFT: East London Foundation Trust
SD: Standard Deviation
SOLES: Sign O’Brien Level of Engagement Scale
